# Out of sight, out of mind? Germ cells and the potential impacts of epigenomic drugs

**DOI:** 10.12688/f1000research.15935.1

**Published:** 2018-12-21

**Authors:** Ellen G. Jarred, Heidi Bildsoe, Patrick S. Western

**Affiliations:** 1 Centre for Reproductive Health, Hudson Institute of Medical Research, Clayton, Victoria, 3168, Australia; 2 Department of Molecular and Translational Science, Monash University, Clayton, Victoria, 3168, Australia

**Keywords:** epigenetic, inheritance, development, germline, pharmacology, cancer, reproduction

## Abstract

Epigenetic modifications, including DNA methylation and histone modifications, determine the way DNA is packaged within the nucleus and regulate cell-specific gene expression. The heritability of these modifications provides a memory of cell identity and function. Common dysregulation of epigenetic modifications in cancer has driven substantial interest in the development of epigenetic modifying drugs. Although these drugs have the potential to be highly beneficial for patients, they act systemically and may have “off-target” effects in other cells such as the patients’ sperm or eggs. This review discusses the potential for epigenomic drugs to impact on the germline epigenome and subsequent offspring and aims to foster further examination into the possible effects of these drugs on gametes. Ultimately, the information gained by further research may improve the clinical guidelines for the use of such drugs in patients of reproductive age.

## Introduction

Sperm and oocytes (eggs) occupy a unique position in biology, as they transmit genetic and epigenetic information from parent to offspring in sexually reproducing organisms. An individual’s genes provide the primary genetic information that determines phenotypic outcomes in our children—whether they have blue eyes, are suited to sprinting or long distance running, or will be susceptible to certain diseases, and so on. While the DNA contains the primary genetic sequence, chemical modifications to the DNA and associated histone proteins influence how the DNA is organised within the nucleus and whether specific genes are switched on or off. This epigenetic information is critically important for the interpretation of the DNA during development of the foetus and in adult life, strongly influencing cell specification, phenotypic outcomes, and adult health. Moreover, epigenetic modifications are heritable, ensuring that a memory of cell-specific gene activity is transmitted during cell division, facilitating cell and tissue function. Here, we consider epigenetic modifications to include DNA methylation and histone modifications that are mitotically or meiotically stable (or both) and contribute to cellular memory. As the term “epigenetic” has been more broadly interpreted, more in-depth discussions can be found in recent stimulating reviews from Steven Henikoff and John Greally ^1, 2^.

In addition to regulating cell-specific gene expression profiles, epigenetic mechanisms provide a potential interface between the environment and genomic function, including in the germline. Changes mediated by environmental influences, such as diet, drugs, or chemicals, are thought to alter epigenetic programming in germ cells, resulting in epigenetic differences in sperm and eggs that may alter outcomes in offspring (reviewed in 3– 9). In this context, examples of environmental factors are provided by epigenetic modifying drugs, which have attracted substantial interest in oncology but have been studied in only very limited detail with respect to their impacts on germline epigenetic programming and epigenetic inheritance. In the context of this discussion, “epigenomic drugs” include pharmaceuticals that specifically alter the activity of enzymes or proteins that mediate DNA methylation and histone modifications. Although these drugs have great potential for improving clinical outcomes in patients, they may also directly alter the germline epigenome and potentially have deleterious outcomes for future offspring.

Despite a substantial number of studies examining the impacts of diet and other environmental effects on the germline epigenome and inheritance, the potential impacts of epigenomic therapies on the germline have been largely “off the radar” when assessing drug impacts on patients. This is likely due to the primary focus of clinical trials and treatment on safety and improving patient health, whereas reproductive and offspring health are usually secondary considerations. Clearly, these primary aspects of therapy are of paramount importance, and effective therapies should be used to ensure the best possible outcomes for patients. The purpose of this review is not to vilify epigenomic drugs or to discourage their use by patients or prescription by clinicians. However, given the potential impacts of the germline epigenome on offspring, it is important that future research aims to understand how these drugs might change the germline epigenome and whether such changes affect offspring development and health. In the long term, this information may facilitate the development of guidelines and pre-treatment advice for patients with respect to future reproduction and, if required, recommendations for fertility preservation.

## Germline epigenetics: programming outcomes in future offspring

The potential for environmental agents to alter the germline epigenome and offspring phenotype has driven a range of studies in germline development and epigenetics. The current conceptual framework for epigenetic inheritance in mammals is dominated by our understanding of DNA methylation, particularly genomic imprinting, which has been intensively studied since its discovery 35 years ago ^10– 12^. Genomic imprinting involves the differential DNA methylation of the paternal or maternal allele of over 120 genes in the developing male or female germlines, resulting in parent-specific epigenetic regulation of these genes in offspring and as a consequence impact on a range of physiological and behavioural outcomes (reviewed in 12– 15). However, recent studies have revealed that other epigenetic modifications in sperm and oocytes can also influence outcomes in offspring, including histone modifications and associated non-coding RNAs ^16– 28^. Some examples include impacts on histone 3 lysine 4 (H3K4) ^22^ and H3K27 methylation ^20^ and DNA methylation-independent imprinting mediated by methylation of H3K27 in the oocyte ^21^. Furthermore, interactions among histone modifications, DNA methylation, and other interacting molecules, such as non-coding RNAs, add complexity to the mechanisms mediating heritable outcomes in offspring ^29, 30^. Such interactions are likely to underlie organised retention and patterning of modified histones and DNA methylation in sperm ^31– 35^ and the potential for environmental challenges, such as diet, chemicals, and drugs, to interact with the germline epigenome and alter paternal inheritance ^16, 36^. Although these and other studies are making substantial progress in understanding germline epigenetics and inheritance, much remains to be discovered.

Epigenetic changes in the germline potentially lead to intergenerational or transgenerational impacts on offspring ^37, 38^, and understanding these differences is important for determining the persistence of potential epigenetic changes induced in germ cells. Intergenerational inheritance occurs when the effect of an environmental stressor is transmitted from a parent (the F0 generation) to their offspring (the F1 generation). In the case of *in utero* exposure, the germ cells of the exposed foetus (F1) ultimately give rise to the F2 generation; therefore, effects transmitted from the F1 foetus to F2 offspring are considered intergenerational, as both the F0 and F1 generations were directly exposed to the environmental agent. Transgenerational inheritance occurs when an effect persists in the absence of direct germline exposure. For example, when the germline of the F0 parent is exposed, effects detected in the F2 generation can be considered transgenerational. Similarly, effects transmitted following *in utero* exposure of the F1 foetus that are detected in the F3 generation are considered transgenerational ^37, 38^. Importantly, these effects must survive epigenetic reprogramming in the F1 or F2 germline, respectively, to be transmitted transgenerationally ^38^.

**Figure 1.**
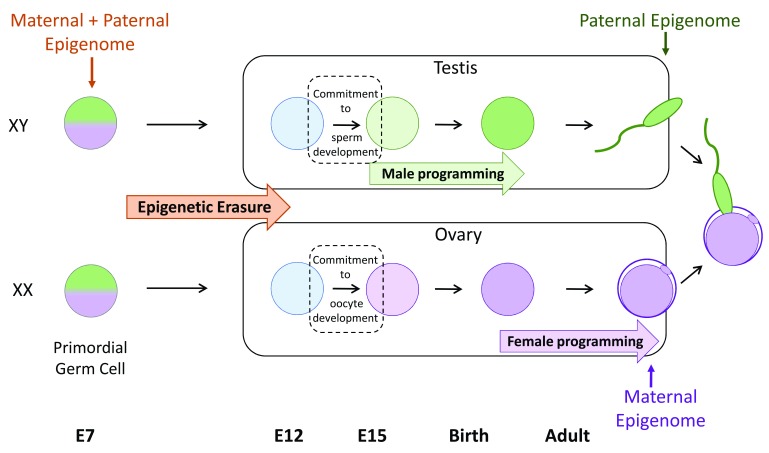
Primordial germ cells undergo extensive epigenetic reprogramming prior to transmitting epigenetic information to offspring. Primordial germ cells (PGCs) are the earliest precursors of sperm and oocytes (eggs) and are specified in the mouse around embryonic day 7 (E7). Initially, PGCs carry both paternal (green) and maternal (purple) epigenetic modifications that are similar to the somatic cells from which PGCs are derived. This information is removed by a process of epigenetic erasure before sex-specific epigenomes (paternal in male germ cells and maternal in female germ cells) are established. Epigenetic erasure occurs as PGCs migrate towards and populate the developing gonads. Soon after reaching the developing testis or ovary, germ cells commit to spermatogenesis or oogenesis, respectively. Subsequently, sex-specific epigenetic information is established. This occurs at different stages of development for males and females: during late foetal stages and early post-natal life in males and during oocyte growth in adult females. This results in the production of gametes which are epigenetically non-equivalent but which contain complementary epigenetic information at fertilisation.

As with epigenetic inheritance, epigenetic reprogramming in the germline is currently best understood from the perspective of changes in DNA methylation. Two distinct reprogramming events occur in mice: the first occurs in newly specified primordial germ cells (PGCs) during mid-gestation embryo development, and the latter occurs in the preimplantation embryo ^39^. In mice, PGCs are specified from surrounding epiblast cells at embryonic day 7 (E7) ^40– 42^ and around week 2 to 3 in humans ^43^. Initially, the newly specified PGCs carry epigenetic modifications similar to those of their somatic precursors, including epigenetic modifications from both parents. These epigenetic modifications are incompatible with sex-specific epigenetic programming and must be removed from PGCs before new, parent-specific (that is, only maternal or paternal) modifications that are compatible with offspring development can be established ( Figure 1). Reprogramming in the female and male germlines allows the production of oocytes and sperm that contain maternal- and paternal-specific information that is complementary at fertilisation and supports normal offspring development. Moreover, reprogramming in the germline facilitates the resetting of epigenetic errors that may have accumulated in the germline, preventing transmission of these altered epigenetic states to offspring. Fertilisation initiates the second reprogramming event, which establishes developmental competence in the preimplantation embryo, partly by resetting the maternal and paternal genomes to functional equivalence for many developmental genes. However, preimplantation epigenetic reprogramming leaves inherited epigenetic modifications such as parent-specific genomic imprints intact ^44– 50^, allowing their parent-specific function later in life. This represents a key difference between the two reprogramming events: reprogramming in PGCs occurs in order to remove existing genomic imprints and other epigenetic information, whereas reprogramming in preimplantation embryos occurs to establish equivalence between the paternal and maternal genomes and restore totipotency but with the exception that genomic imprints and possibly other parent-specific epigenetic information are maintained rather than lost.

Germline epigenetic reprogramming is achieved during PGC migration and final settlement of germ cells in the developing gonads by E10.5 in mice ^51, 52^ and around week 6 in humans ^43^. The PGCs undergo extensive global DNA demethylation while migrating to the genital ridge, followed by further demethylation as germ cells colonise the developing gonads (the future testes/ovaries). This results in global DNA methylation levels being reduced from around 70% in germline precursor cells to 14% and 7% in male and female PGCs by the time early testes and ovaries have formed at E13.5 and includes loss of methylation at imprinted regions ^53^. Global DNA demethylation is likely to occur via both passive and active mechanisms. Whereas passive loss involves the gradual dilution of methylated cytosine during replication in the absence of maintenance DNA methyltransferase 1 (DNMT1) activity ^54^, active demethylation involves the conversion of 5-methylcytosine to 5-hydroxymethylcytosine by ten-eleven translocase (TET) enzymes TET1 and TET2 and subsequent base excision repair ^55, 56^. Recent work demonstrated that TET1 alone is not essential for DNA demethylation in PGCs, indicating that TETs may act redundantly in this system or that TET1 is primarily required to maintain, rather than drive, DNA demethylation in PGCs ^57^. However, TET1 is required for the activation of germline development genes during reprogramming, indicating that DNA demethylation at specific genes is important for sex-specific germline development ^57^.

After removal, DNA methylation must be re-established in a sex-specific pattern in the male and female germlines. This occurs at quite different times in male and female mice. In male germ cells, new DNA methylation is established at paternally imprinted genes and repetitive sequences by the action of the *de novo* DNA methyltransferase DNMT3A and the co-factor DNMT3L during foetal life ^58, 59^. However, substantial remodelling also occurs during spermatogenesis in adult life, including changes in DNA methylation as germ cells enter meiosis ^60^ (reviewed by 61). In females, new DNA methylation including maternal imprints is established post-natally in oocytes. This occurs in each oocyte after individual primordial follicles have been released from the follicle reserve and progress through an extended growth phase that culminates in oocyte maturation ^27, 58, 62– 64^.

Extensive chromatin remodelling also occurs on histones during germline reprogramming. When specified, PGCs are enriched with the repressive modification H3K9me2. However, during germ cell migration, H3K9me2 is replaced with an alternative repressive modification, H3K27me3 ^65, 66^. Further removal or reorganisation of repressive histone modifications or both occur once germ cells enter the developing gonad ^65– 68^. Although the mechanisms and biological significance of these changes are yet to be determined, H3K27me3 is established at developmental genes in germ cells and repetitive sequences during foetal life ^33, 69^ and is also present at developmental genes in sperm, indicating an important role for this modification in the paternal germline and, potentially, offspring ^69, 70^. Consistent with this, the complex required for catalysing H3K27me3, PRC2, is required for spermatogenesis and male fertility ^71, 72^. Moreover, recent work has demonstrated a role for PRC2 in epigenetic programming in foetal male germ cells and in modulating paternal epigenetic inheritance ^73^. Although H3K27me3 is not essential for female fertility, it is enriched in growing oocytes and PRC2 is required for regulating maternal inheritance ^74^. Deletion of the PRC2 genes *Ezh2* or *Eed* specifically from the growing oocyte resulted in offspring with altered birth weights ^20, 75^, bone mineral density, and fat and muscle content and reduced litter size ^20^. In addition, H3K27me3 is required for regulating a non-coding RNA and consequently genomic imprints in mice ^29^, and overexpression of the histone demethylase *Kdm6b* in the zygote revealed a role for H3K27me3 in DNA methylation-independent imprinting ^21^. Similarly, the H3K4 methylase SETD1B regulates oocyte-specific RNAs ^76^, and increased levels of H3K4me2 in sperm resulted in paternally transmitted developmental effects in mice ^22^.

These examples demonstrate the importance of a range of epigenetic mechanisms in the male and female germlines, but they generally do not identify when the germline is most vulnerable to epigenetic change or how specific environmental agents impact on the germline. Given the differences in the timing of sex-specific DNA methylation and establishment of imprints, the periods of greatest sensitivity to environmentally induced epigenetic change may also differ in male and female germ cells. For example, male germ cells may be most vulnerable during foetal life whereas female germ cells may be most vulnerable during oocyte growth in adults. However, this does not exclude changes at other stages, such as during the extensive nuclear remodelling and histone replacement/rearrangement that occur during spermatogenesis or within the follicle reserve that contains the oocytes that underpin the reproductive life of females. Understanding epigenetic programming in both mechanistic and temporal frameworks will help illuminate the stages during which the germline is most sensitive to specific environmental factors and the potential risks of different exposures.

## Emerging environmental agents: could epigenomic drugs affect the germline epigenome and future offspring?

**Figure 2.**
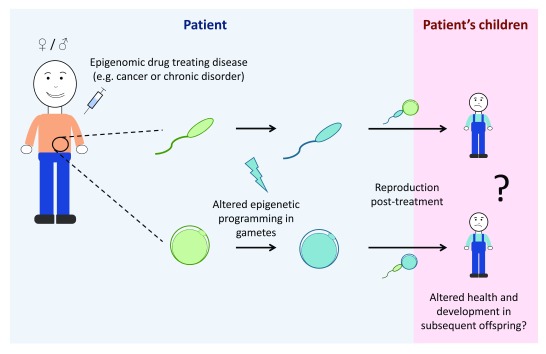
Epigenomic drugs may alter epigenetic programming in the germline and may alter health and development in offspring. Epigenomic drugs are being used for cancer therapies and other disorders such as epilepsy; however, potential impacts of epigenomic drugs on the germline remain largely unexplored. As germ cells contain substantial epigenetic information, treatment with epigenomic drugs may alter the epigenetic information in sperm and oocytes (eggs). As epigenomic drugs work systemically, changes to the germline epigenome cannot be excluded and may result in altered health and development of subsequent offspring. In this diagram, the pale-blue background represents what is occurring in the patient whereas the pink background represents the patient’s children. Green gametes represent epigenetically normal sperm and oocytes, whereas blue gametes represent sperm and oocytes with altered epigenomes.

Although many studies indicate that a large range of environmental stimuli may affect the germline epigenome and consequently offspring phenotype, the underlying mechanisms are often poorly understood (reviewed in 3– 9). One relatively obvious way that epigenetic programming in the germline could be altered is through the action of agents that directly inhibit the enzymes that mediate epigenetic change. Indeed, the dynamic nature of epigenetic modifications coupled with the prevalence of dysregulated epigenetic modifying enzymes in tumours has led to the development of an extensive range of pharmacological inhibitors of specific epigenetic modifying enzymes for cancer therapies ^77, 78^. In the context of oncology, these drugs are being used to either kill cancer cells or drive their differentiation. It has been estimated that approximately half of all tumours involve abnormalities in chromatin modifier proteins (including epigenetic modifiers), and substantial efforts in pharmacological science are directed towards developing therapeutics for as many of these chromatin modifiers as possible (reviewed in 77, 78). This area of pharmacology is rapidly expanding, and these drugs offer highly promising new therapies that are clearly important for patients. However, these drugs act systemically and their potential impacts on the germline and future offspring remain largely unexplored. A number of studies have addressed whether epigenomic drugs alter the germline epigenome but have tended to focus on direct impacts on fertility rather than outcomes in offspring as a result of germline exposure to the drug ( Table 1). Moreover, although some studies have tested clinically relevant drug doses in mice, the clinical relevance of doses used in other studies has not always been clear. As differing drug doses are expected to affect target epigenetic modifications to varying degrees, it is important to test doses that reflect those used in humans as closely as possible when determining germline drug impacts in model organisms. Despite these challenges, it is important to explore the potential impacts of these therapies on the germline and subsequent offspring to generate a greater knowledge base from which clinical guidelines can be developed for the use of epigenomic drugs in children or patients of reproductive age ( Figure 2).

**Table 1.** Studies of intergenerational or transgenerational impacts of epigenomic drugs *in utero* or in adults.

Inhibitor type and mechanism	Drug	Target	Approval/trial status	Studies in germline	Characteristics assessed	Studies in offspring	Characteristics assessed
**DNMTi** **Inhibit DNA** **methyltransferases**	Azacytidine (Vidaza)	Pan- DNMT	FDA	Zhao *et al*. (2013) ^79^ Doerksen and Trasler (1996) ^80^ Seifertová *et al.* (1976) ^81^	Oocyte *in vitro* maturation rate and chromosome condensation ^79^ Male fertility ^80, 81^	Doerksen and Trasler (1996) ^80^	Pre- and post- implantation development of embryos of treated males (embryo loss, litter size, and gross morphology) ^80^ Embryo loss following treatment of adult males ^81^
	Decitabine (Dacogen)	Pan- DNMT	FDA	Kelly *et al*. (2003) ^82^ Oakes *et al*. (2007) ^83^ Kläver *et al*. (2015) ^84^ Song *et al.* (2016) ^85^ Cisneros and Branch (2003) ^86^ Raman *et al.* (1995) ^87^	Male fertility and global DNA methylation in sperm ^82, 83^. DNA methylation at paternally imprinted regions in sperm ^83^ Male fertility ^84^ Male fertility (sperm production only) ^85^ Fertility of *in utero* exposed males and females ^86^ Male fertility (sperm production only) ^87^	Kelly *et al*. (2003) ^82^ Oakes *et al*. (2007) ^83^ Kläver *et al.* (2015) ^84^	Pre- and post- implantation development of embryos of treated males (embryo loss, litter size, and gross morphology) ^82^ Progression through pre-implantation development *in vitro* ^83^ Fertility in male offspring of treated males ^84^
	Guadecitabine	Pan-DNMT	Phase I, II, and III	None		None	
**HDACi** **Inhibit histone** **deacetylases**	Belinostat (Beleodaq)	HDAC class I and class II	FDA	None		None	
	Panobinostat (Farydak)	HDAC class I, II, and IV	FDA	None		None	
	Romidepsin (Istodax)	HDAC class I	FDA	None		None	
	Vorinostat (Zolinza)	HDAC class I, II, and IV	FDA	Wise *et al.* (2008) ^88^ Kläver *et al.* (2015) ^84^	Male and female fertility and pregnancy outcome ^88^ Male fertility ^84^	Kläver *et al.* (2015) ^84^	Fertility in male offspring of treated males ^84^
	Valproic acid	HDAC class I and II	FDA/phase III	Bairy *et al.* (2010) ^89^ Røste *et al.* (2001a) ^90^ Røste *et al.* (2001b) ^91^ Røste *et al*. (2003) ^92^	Male fertility (sperm production only) ^89^ Effects of valproate on ovarian morphology ^90^ Male reproductive system and fertility ^91^ Semen parameters in male epilepsy patients taking valproic acid ^92^	Choi *et al.* (2016) ^93^	Transgenerational inheritance of autism- like behaviours following *in utero* exposure ^93^
	Tacedinaline (CI994)	HDAC class I, II, III, and VIII	Phase III	None		None	
	HDACi 4b [N1-(20aminophenyl)-N7- phenoylheltanediamide]	HDAC class I, II, and III	Not in clinic	None		Jia *et al.* (2015) ^94^	Intergenerational effects in Huntington’s disease ^94^
**BET inhibitors** Inhibit BET domain family proteins	Apabetalone/RVX000222/RVX-208	Pan-BET	Phase III	None		None	
	JQ1	Pan-BET	Not in clinic	Matzuk *et al.* (2012) ^95^	Male fertility ^95^	Matzuk *et al.* (2013) ^95^	Production of offspring from treated males ^95^
	CPI-0610	Pan-BET	Phase I	None		None	
	TEN-010	Pan-BET	Phase I	None		None	
	BAY1238097	Pan-BET	Phase I	None		None	
	OTX015	Pan-BET	Phase I	None		None	
	INCB054329	Pan-BET	Phase I and II	None		None	
	BMS-986158	Pan-BET	Phase I and II	None		None	
	FT-1101	Pan-BET	Phase I and II	None		None	
	GSK525762	Pan-BET	Phase I	None		None	
**LSD1 inhibitors** Inhibit histone demethylase LSD1	GSK2879552	LSD1	Phase I	None		None	
	IMG-7289	LSD1	Phase I	None		None	
	INCB059872	LSD1	Phase I	None		None	
	Tranylcypromine	LSD1	Phase I	None		None	
**EZH2 inhibitors** Inhibit histone methyltransferase EZH2	Tazemetostat	EZH2	Phase I and II	Prokopuk *et al.* (2018) ^74^	H3K27me3 levels in oocytes and foetal germ cells following drug treatment and withdrawal ^74^	None	
	CP1-1205	EZH2	Phase 1	None		None	
	GSK126	EZH2	Not in clinic	Prokopuk *et al.* (2017) ^68^	H3K27me3 dynamics during epigenetic reprogramming in foetal germ cells ^68^	None	
	UNC-1999	EZH2	Not in clinic	None		None	
	EBI-2511	EZH2	Not in clinic	None		None	

We have included published articles investigating the effects of post-natal exposure to epigenomic drugs on the germline or subsequent offspring or both. Studies assessing *in utero* exposure were included if outcomes were assessed in the germline or in the offspring of exposed foetuses. Direct teratogenic effects were excluded. Table modified from Jones *et al*. ^77^ to form the basis of the drug list, and additional drugs were added. This drug list is extensive though not exhaustive. BET, bromodomain extra terminal; DNMT, DNA methyltransferase; DNMTi, DNA methyltransferase inhibitor; EZH2, enhancer of zeste 2; FDA, US Food and Drug Administration; H3K27me3, H3K27 trimethylation; HDAC, histone deacetylase; HDACi, histone deacetylase inhibitor.

Impacts of epigenomic drugs may alter reproductive health in a range of ways. From a germline perspective, perhaps the most obvious are the potential impacts on fertility, including zygote (fertilised egg) and early embryo viability. For example, rats treated with the class I/II histone deacetylase (HDAC) inhibitor vorinostat were fertile, but increased peri- and post-implantation embryo loss was observed in treated females crossed with untreated males ^88^. Furthermore, although some offspring survived, there was no analysis of their developmental outcomes, so it remains unclear whether treatment of adult females resulted in developmental differences in offspring. In another example, increased ovarian cysts and decreased corpora lutea were observed in female rats treated with an alternative HDAC inhibitor, valproic acid (VPA) ^90^. Mechanistically, extensive HDAC-dependent histone deacetylation occurs during oocyte maturation in mice, which can be blocked by treatment with the HDAC inhibitor trichostatin A ^96^. Moreover, genetic ablation of both *Hdac1* and *Hdac2* in growing oocytes resulted in the arrest of follicle development at the secondary follicle stage ^97^. In addition, VPA affected male fertility in rats and had mild effects in male patients undergoing long-term treatment for epilepsy ^91, 92^. Whereas these studies largely focussed on fertility and embryo viability, some studies have examined the impacts of HDAC inhibitor treatment in paternal inheritance. In a model of Huntington’s disease (HD), the treatment of adult F0 mice altered DNA methylation patterns in sperm and ameliorated disease phenotype in F1 offspring, apparently through a mechanism involving histone demethylation and DNA methylation ^94^. Although these effects potentially imparted some beneficial intergenerational effects on behaviour in this HD model, the broader impacts on offspring health were not examined. This is an important point, as impacts of this drug on DNA methylation and histone methylation, as well as histone acetylation, strongly indicate that the effects of treatment were unlikely to be focussed only on HD genes but are likely to have altered other aspects of inheritance. Indeed, the treatment of pregnant female mice with VPA led to the inducement of autism-like behaviours not only in the directly exposed F1 offspring but also in the unexposed F2 and F3 offspring of exposed F1 progeny, demonstrating a detrimental transgenerational effect of VPA in inheritance ^93^.

While DNA methylation is the best-understood marker of inherited epigenetic modifications, there is limited understanding of the impacts of DNMT inhibitors on the female germline and inheritance. In oocytes matured *in vitro* in the presence of azacytidine (Vidaza), chromosomes were less condensed and more unstable than in untreated controls ^79^. Although treatment induced the expression of early apoptotic markers, these oocytes progressed through maturation faster than did untreated controls, and it was concluded that azacytidine treatment imparted a beneficial effect ^79^. However, the potential for the resulting oocytes to be fertilised or to support normal offspring development was not assessed. In another study, *in utero* decitabine (Dacagon)-exposed females mated with untreated males had normal fertility and produced offspring of normal weights ^86^, but other potential impacts on offspring outcomes were not assessed.

A range of studies in males have demonstrated that both azacytidine and decitabine impair spermatogenesis or male fertility or both, and some reported reduced litter size or embryo loss or both ^80– 87^. Although these treatments reduced male fertility, this effect was reversible when treatment was withdrawn. In one study male mice restored normal testis histology ^85^, and in another study drug-induced foetal loss was prevented ^81^ 4 weeks after treatment was terminated. Moreover, although subtle effects on reproductive organs and sperm parameters were observed in F1–F3 offspring of treated males, neither decitabine nor vorinostat caused intergenerational or transgenerational effects on male fertility ^84^. Although these studies demonstrate substantial, though reversible, impacts of DNMT inhibitors on male fertility and litter size, the broader impact of these drugs on developmental outcomes in offspring remains largely unknown.

DNA methyltransferase and HDAC inhibitors are well established in the clinic and have been used in a range of combination therapies, and their actions in relation to cancer treatment are becoming better understood ^77, 98– 103^. However, more recent developments have focussed on histone methyltransferase (HMT) and bromodomain extra terminal repeat (BET) inhibitors ^104– 107^ ( Table 1). Very few studies have examined the impacts of BET inhibitors on the germline, although the preclinical BET inhibitor JQ1 has been proposed as a potential male contraceptive because of its ability to reversibly block male fertility ^95^. As with DNMT inhibitors, withdrawal of JQ1 treatment restored fertility and these males produced apparently normal pups ^95^, but developmental outcomes in these offspring were not assessed in detail.

**Figure 3.**
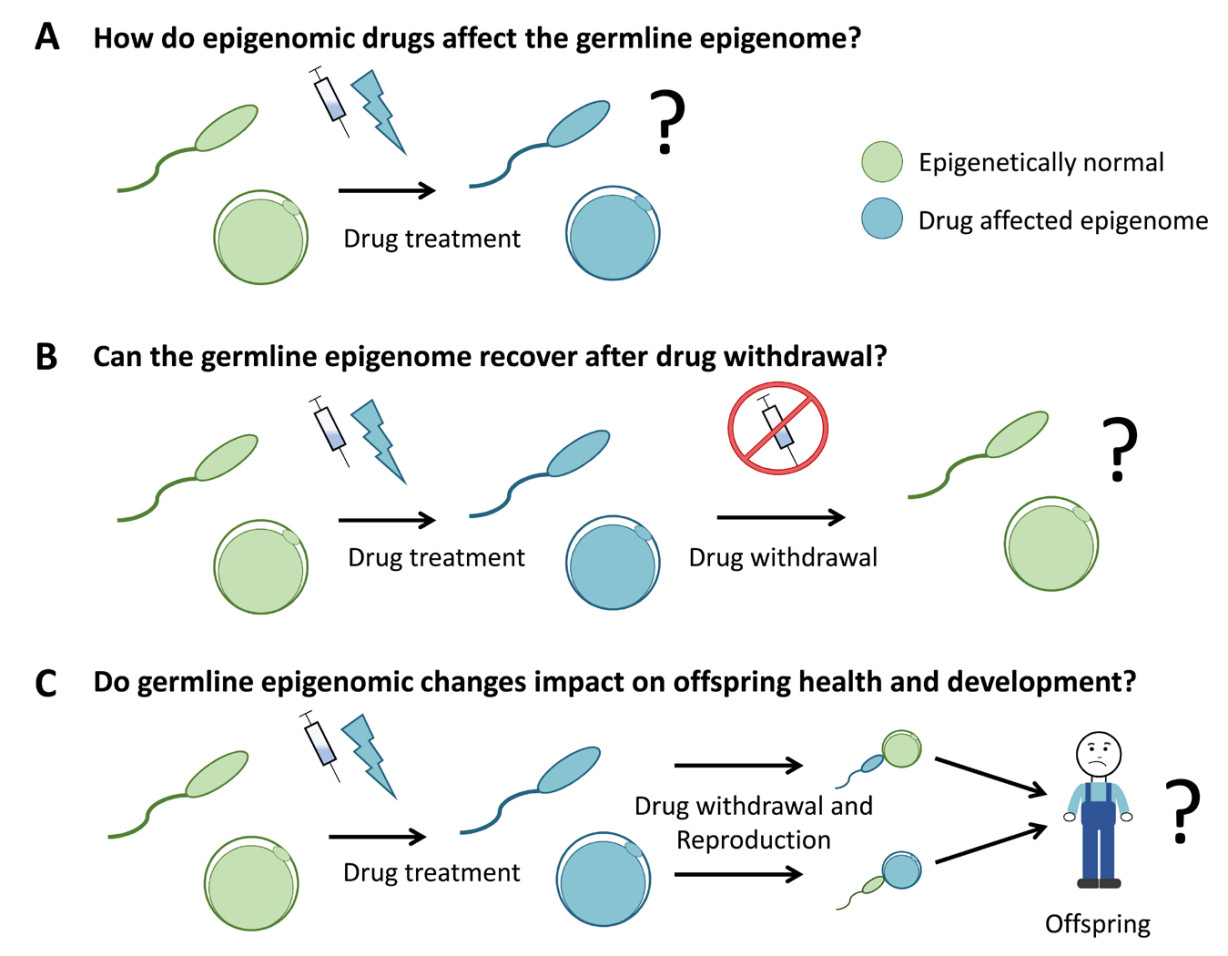
The potential for epigenomic drugs to impact the germline remains largely unexplored. Three key areas of investigation for specific drugs are understanding ( **A**) the capacity of epigenomic drugs to affect the germline epigenome, ( **B**) the capacity of the germline to recover after drug withdrawal, and ( **C**) whether changes induced in the germline epigenome impact on the health and development of subsequent offspring. Collectively, this information will aid in refining clinical guidelines for the use of epigenomic drugs in patients of reproductive age and in children/adolescents prior to reproduction. In this diagram, green gametes represent epigenetically normal sperm and oocytes (eggs) and blue gametes represent sperm and oocytes with altered epigenomes.

Another prominent epigenomic target is the H3K27 histone methyltransferase EZH2, for which drugs include EPZ-6438 (tazemetostat), GSK126, CPI-1205, EBI-2511, and UNC1999 ^108– 112^ ( Table 1). Perhaps the most clinically advanced of these is tazemetostat, which is being assessed in a range of phase I/II clinical trials for human patients presenting with a variety of cancers, including lymphomas, myelomas, mesothelioma, solid tumours, and malignant rhabdoid tumours of the kidney and ovary ( http://clinicaltrials.gov). Patient cohorts include individuals of reproductive age and children as young as 6 months. Recent work demonstrated that the treatment of adult female mice with a clinically relevant dose of tazemetostat for 10 days significantly depleted H3K27me3 in growing oocytes, and H3K27me3 did not recover after a 10-day period of drug withdrawal ^74^. This is concerning given that oocyte-specific deletion of *Ehz2* caused growth restriction in offspring ^20, 75^ and similar oocyte-specific deletion of the essential *Ezh2*-interacting gene *Eed* caused foetal overgrowth, increased bone mineral density, and altered fat and muscle content in offspring ^20^. Similarly, recent work demonstrated a role for PRC2 in regulating paternal epigenetic inheritance ^73^. Moreover, *de novo* germline mutations in the PRC2-encoding genes *EZH2, EED*, or *SUZ12* in humans result in Weaver and Cohen–Gibson syndromes, characterised by a spectrum of abnormalities including over-growth, skeletal defects, and cognitive deficits ^113– 119^. Although these mutations are genetic, the observed phenotypes may have an epigenetic basis. Therefore, given these outcomes, future work should address whether pharmacological depletion of H3K27me3 in growing oocytes can recapitulate the phenotypic outcomes in subsequent offspring observed in the mouse oocyte deletion models or human genetic conditions ^20, 74, 113– 119^. Prokopuk *et al.* also demonstrated depletion of H3K27me3 in the primordial follicle pool of juvenile mouse ovaries cultured with tazemetostat *in vitro* ^74^. As human clinical trials currently include children, it is important to examine whether tazemetostat-induced depletion of H3K27me3 is observed in primordial follicles of juvenile mice *in vivo*, whether H3K27me3 recovers after drug withdrawal, and whether this potential transient loss of H3K27me3 in the oocyte genome affects outcomes in future offspring ( Figure 3).

## Epigenomic drugs during pregnancy

Substantial epigenetic reprogramming occurs during foetal development, making this a period of particular interest for germline exposures to environmental factors. However, as epigenomic drugs target proteins that widely influence specification and development, the use of these drugs during pregnancy is contraindicated in most circumstances.

Despite this, the use of epigenomic drugs in pregnancy is not unprecedented, particularly in the case of ongoing chronic illnesses which require continued treatment during pregnancy. VPA has been used extensively since 1974 to treat both epilepsy and bipolar mania, including in women of reproductive capacity and pregnant women ^120^. However, VPA has been shown to be teratogenic in both animal and human studies, with *in utero* exposure linked to neural tube, cardiac, limb, kidney, craniofacial, and genitourinary defects ^120– 123^. Furthermore, a recent study in mice demonstrated autistic-like behaviours in offspring exposed to VPA *in utero* ^93^. Remarkably, these behaviours were observed in the two subsequent generations, indicating that these effects were maintained transgenerationally ^93^.

The continued use of VPA during pregnancy highlights the difficulty that clinicians and patients face under these conditions: how is the use of a drug that is required by the patient balanced with risk to the unborn child? As the risks of epigenomic drug exposures to the unborn child either through the germline or after direct *in utero* exposure are poorly understood, it is difficult to make informed decisions regarding potential outcomes of such exposures. Animal studies that separate pre-fertilisation from gestational exposures are required to evaluate the underlying mechanisms and relative risks of these two periods.

## Conclusions and considerations for future germlines

Though not exhaustive, the examples described in this review provide a snapshot of the germline epigenome and inhibitors specific to a small group of epigenetic complexes. These studies illustrate the broader concept that a range of epigenetic mechanisms act to establish the germline epigenome and indicate that the effects on the germline by many epigenomic drugs currently under development should be explored. The combined use of both pharmaceutical and genetic models in mice or other animals provides opportunities for well-controlled studies of the impacts of epigenomic drugs and the mechanisms involved in epigenetic inheritance. Furthermore, more human epidemiological studies are also required to evaluate drugs that are currently in use. Key outstanding areas include addressing whether epigenomic drugs alter the germline epigenome, how these changes affect the health and development of subsequent children, and whether these changes are reversed following drug withdrawal ( Figure 3). Outcomes from such studies are required to facilitate more informed clinical use of the drugs with regard to fertility and reproduction and determine whether fertility-preserving approaches should be used to decrease germline exposure to specific drugs or maintain fertility for the patient or both.

## Abbreviations

BET, bromodomain extra terminal repeat; DNMT, DNA methyltransferase; E, embryonic day; EED, embryonic ectoderm; EZH2, enhancer of zeste 2; H3K4, histone 3 lysine 4; H3K9me2, histone 3 lysine 9 dimethylation; H3K27, histone 3 lysine 27; H3K27me3, H3K27 trimethylation; HD, Huntington’s disease; HDAC, histone deacetylase; PGC, primordial germ cell; PRC2, polycomb repressive complex 2; SUZ12, suppressor of zeste 12; TET, ten-eleven translocase enzyme; VPA, valproic acid.
